# Supplementary material for: TiO2-Supported Pd as an Efficient and Stable Catalyst for the Mild Hydrotreatment of Tar-Type Compounds
Source: Nanomaterials (Basel). 2021 Sep 13;11(9):2380. doi: 10.3390/nano11092380 (PMC8471740; doi:10.3390/nano11092380)
Supplement: Supplementary file 1 [file nanomaterials-11-02380-s001.zip › nanomaterials-1337028-supplementary.pdf]

nanomaterials

Supplementary Materials

TiO_2-Supported Pd as an Efficient and Stable Catalyst for the Mild Hydrotreatment of Tar-Type Compounds

Zaher Raad [1,2], Joumana Toufaily [2], Tayssir Hamieh [2] and Marcelo E. Domine [1,*]

[1] Instituto de Tecnología Química (UPV-CSIC), Universitat Politècnica de València, Consejo Superior de Investigaciones Cientificas, Avda. de los Naranjos s/n, 46022 Valencia, Spain; zaraa@upvnet.upv.es
[2] Laboratoire de Matériaux, Catalyse, Environnement et Méthodes Analytiques (MCEMA-CHAMSI), EDST, Université Libanaise, Campus Hariri, Hadath, 1003 Beyrouth, Lebanon; joumana.toufaily@ul.edu.lb (J.T.); tayssir.hamieh@ul.edu.lb (T.H.)
* Correspondence: mdomine@itq.upv.es

Preliminary catalytic screening:

Table S1. Catalytic activity (Conversion and selectivity to the different groups of products) of carbon-supported Pd, Pt and Ru commercial catalysts (comparison at 50–60% conversion).[a.]

Catalyst	Conv. (mol%)	TON [b]	Product selectivity (mol%) [c]							
			MonoAr	Tetralin	Decalin	Ace	HAce-1	HPhe-1	HPhe-2	HAce-2 /HPhe-3
**5wt%Pd/C**	**71**	21	2	32	0	4	42	18	2	0
5wt%Pt/C	55	30	2	29	0	34	13	21	1	0
5wt%Ru/C	52	15	2	32	0	35	14	16	1	0

[a] Reaction conditions: 0.5 g of tars-type compounds, 4 g of n-hexadecane, 0.2 g of catalyst, at 250 °C and 30 bar of H_2 during 7 h. [b] TON = mols of products / mols of metal. [c] Liquid phase composition; Selectivity = mol of product (t) × 100 / total mol of identified products (t).

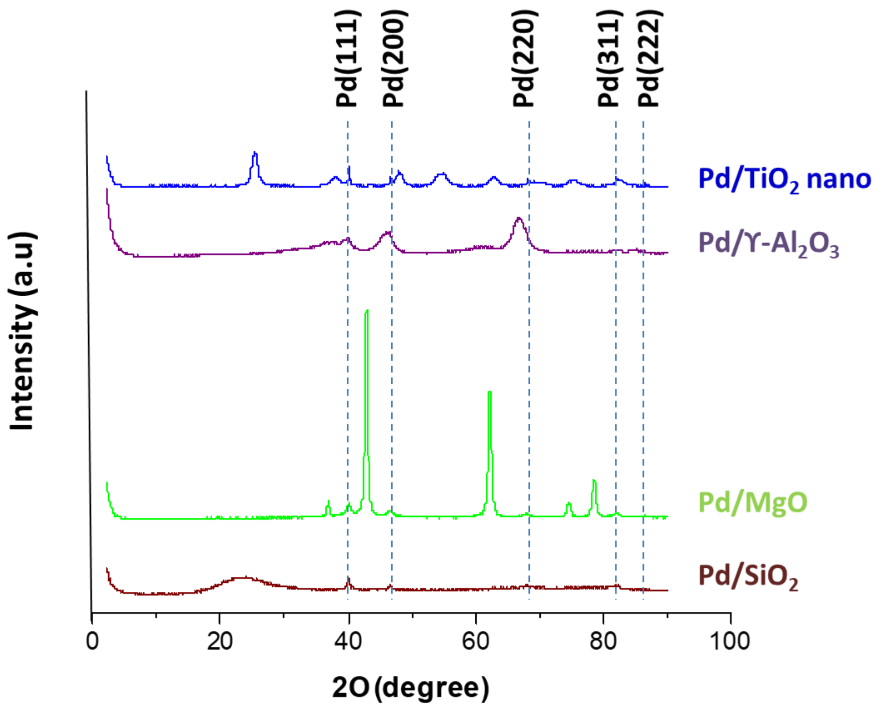

Figure S1. XRD patterns for different metal oxides-supported Pd catalysts.

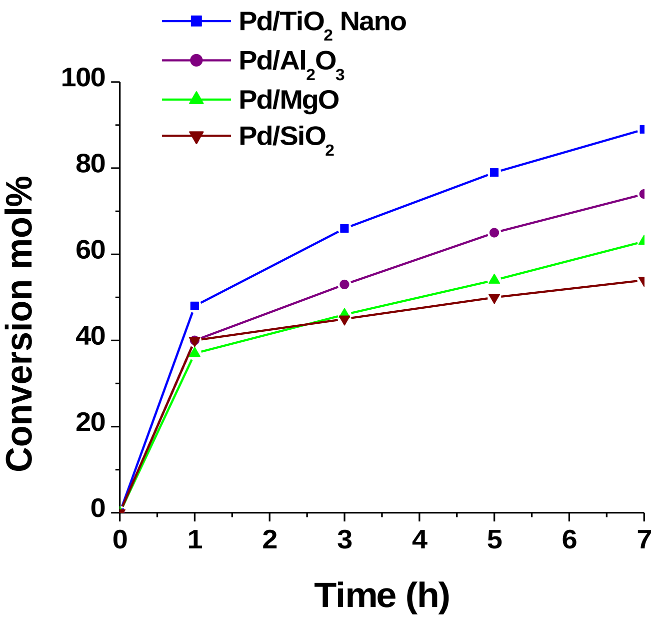

Figure S2. Conversion vs time for different metal oxides-supported Pd catalysts. Reaction conditions: 0.5 g of tar-type compounds, 4 g of n-hexadecane at 250 °C and 30 bar of H_2 during 7 h.

Effect of Pd content in Pd/TiO_2 (Nano) catalysts:

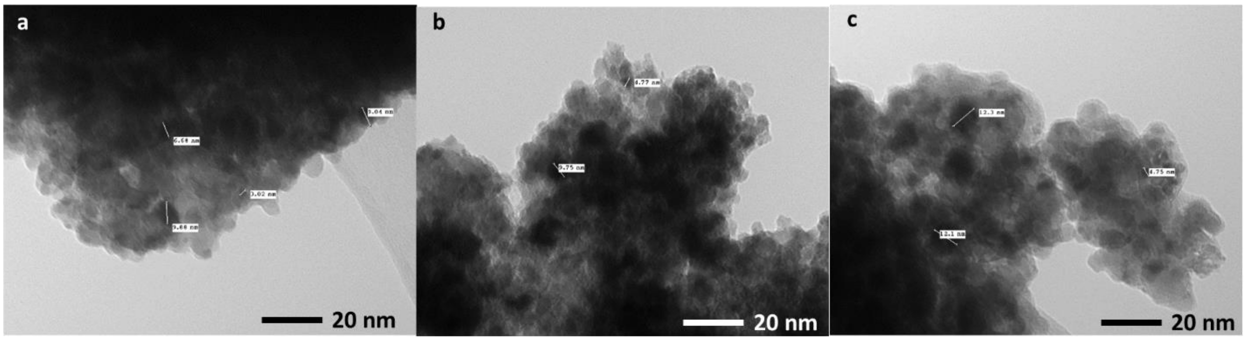

Figure S3. TEM images of (**a**) 0.8wt%Pd/TiO_2 (4–7 nm),(**b**) 1.3wt%Pd/TiO_2 (5–9 nm), and (**c**) 2.2wt% Pd/TiO_2 (6–12 nm).

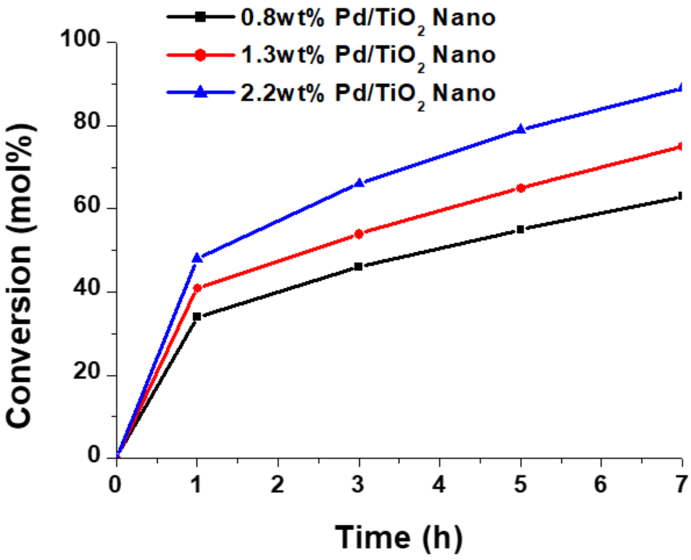

Figure S4. Conversion vs time for Pd/TiO_2 Nano catalysts with different Pd loadings. Reaction conditions: 0.5 g of tars-type compounds, 4 g of n-hexadecane, 0.2 g of catalyst, at 250 °C and 30 bar of H_2 during 7 h.

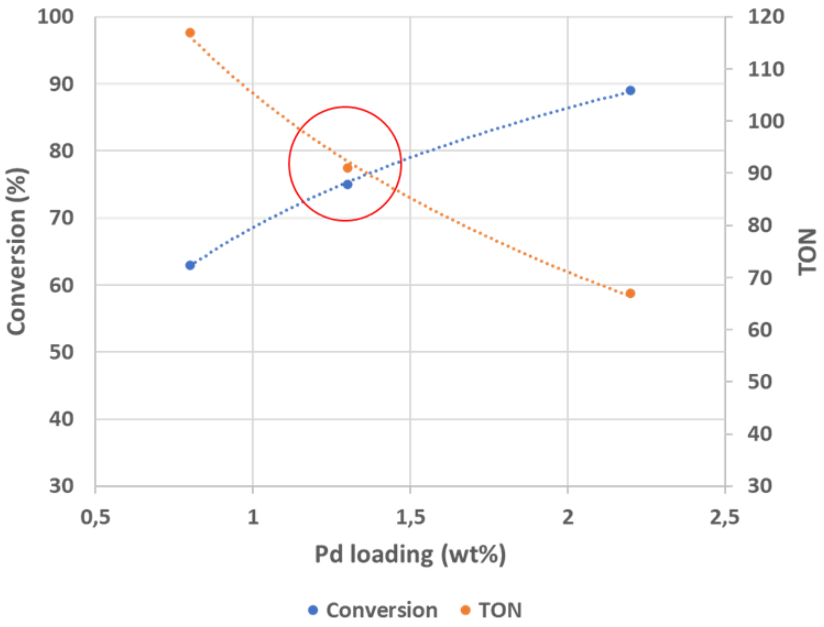

Figure S5. Conversion and TON (mols prod. / mols of Pd in solid) vs Pd loading for Pd/TiO_2 nano catalysts. Reaction conditions: 0.5 g of tars-type compounds, 4 g of n-hexadecane, 0.2 g of catalyst, at 250 °C and 30 bar of H_2 during 7 h.

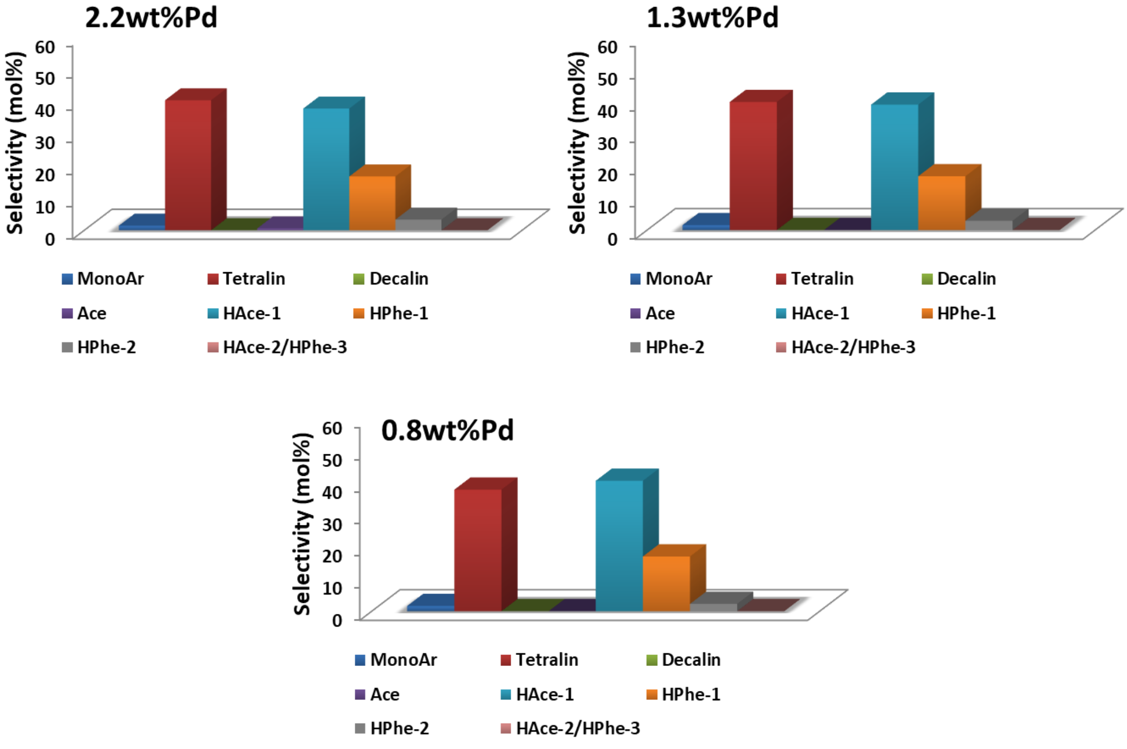

Figure S6. Selectivity to the different groups of products for Pd/TiO_2 Nano catalysts compared in the range 63-65% of conversion. Reaction conditions: 0.5 g of tars-type compounds, 4 g of n-hexadecane, 0.2 g of catalyst, at 250 °C and 30 bar of H_2 during 7 h.

Table S2. Pd/TiO_2 Nano catalysts prepared by using different Pd precursors and their catalytic results in tars mild hydrotreatment.[a.]

Pd Precursor	Pd (wt%)[b]	Conversion (mol%)	TON[c]
$Pd(NH_3)_4.Cl_2.H_2O$	1.3	75	91
$Pd(NO_3)_2.2H_2O$	1.3	79	90
$Pd(NO_3)_2.4NH_3$	0.8	68	127
$Pd(NH_3)_4.Cl_2.H_2O$	0.8	63	117

[a] Reactions conditions: 0.5 g of tars -type compounds, 4 g of n-hexadecane, at 250 °C, 30 bar of $H_{2,}$ 0.2 g catalyst during 7 h. [b]

Measured by ICP; [c] TON = mols of products / mols of metal in solid.

Effect of TiO_2 crystalline phase used as support

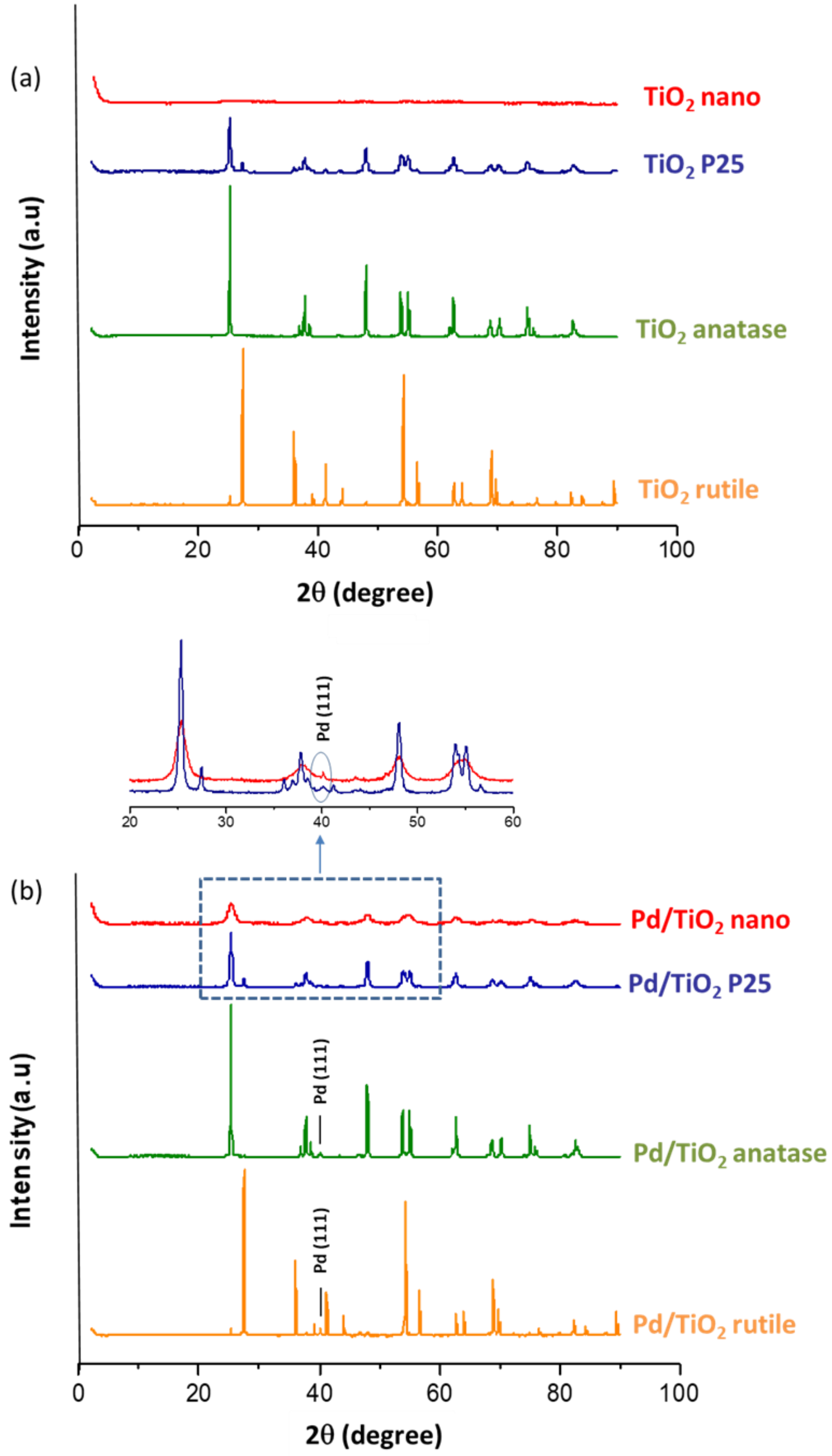

Figure S7. XRD patterns of different (a) pure TiO_2 supports and (b) TiO_2-supported Pd catalysts (Inset showing a zoom of 20-60 2Q region for Pd/TiO_2 nano and Pd/TiO_2 P25).

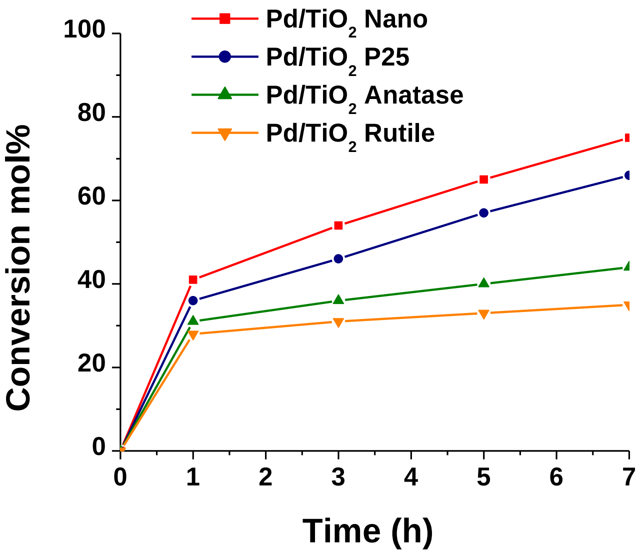

Figure S8. Conversion vs time for different TiO_2-supported Pd catalysts in tars mild hydrotreatment. Reaction conditions: 0.5 g of tars-type compounds, 4 g of n-hexadecane, at 250 °C and 30 bar of H_2 during 7 h.

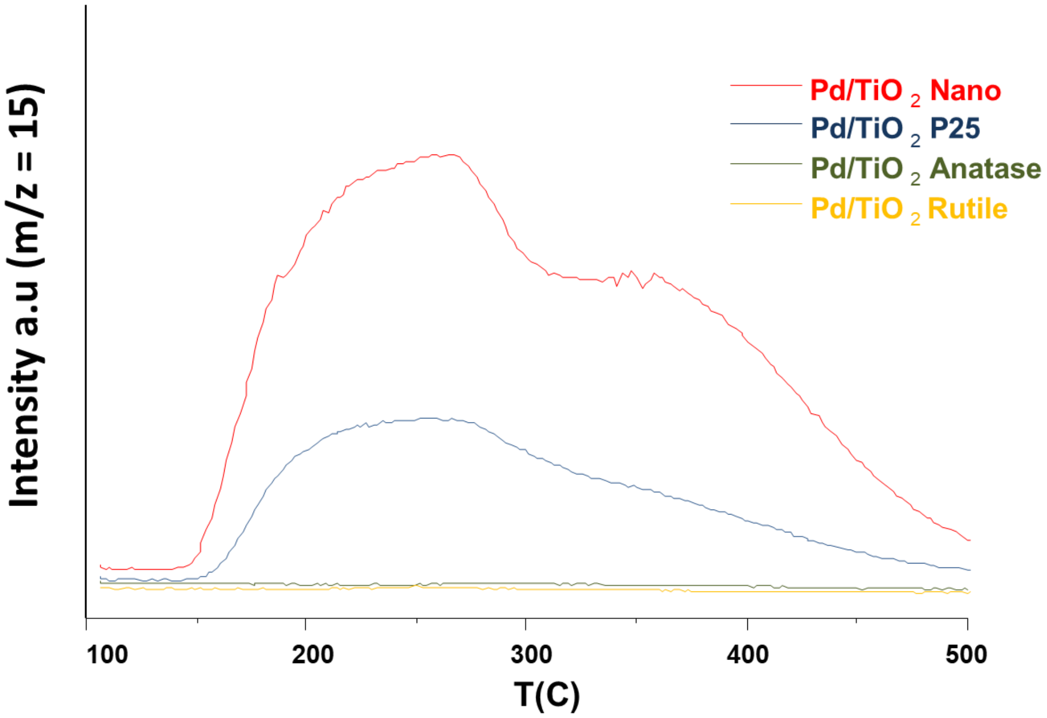

Figure S9. NH_3-TPD profiles for different TiO_2-supported Pd catalysts.

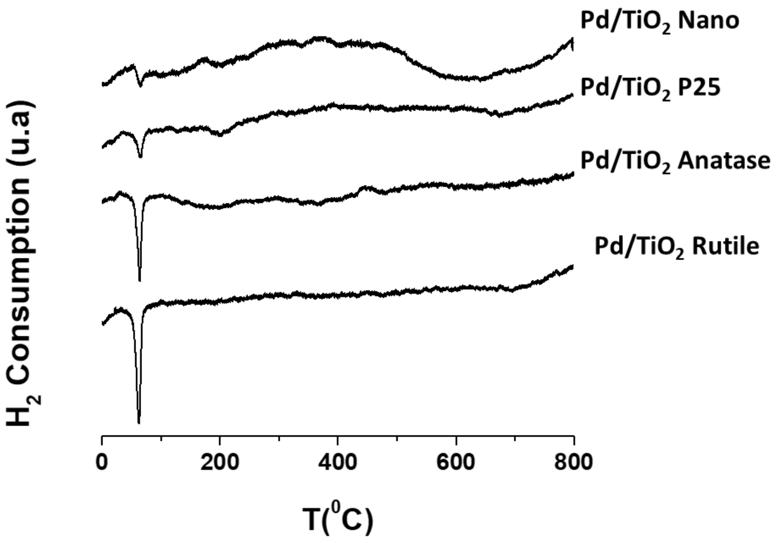

Figure S10. H_2-TPR profiles for different TiO_2-supported Pd catalysts.

Effect of reaction conditions for Pd/TiO_2 Nano catalyst

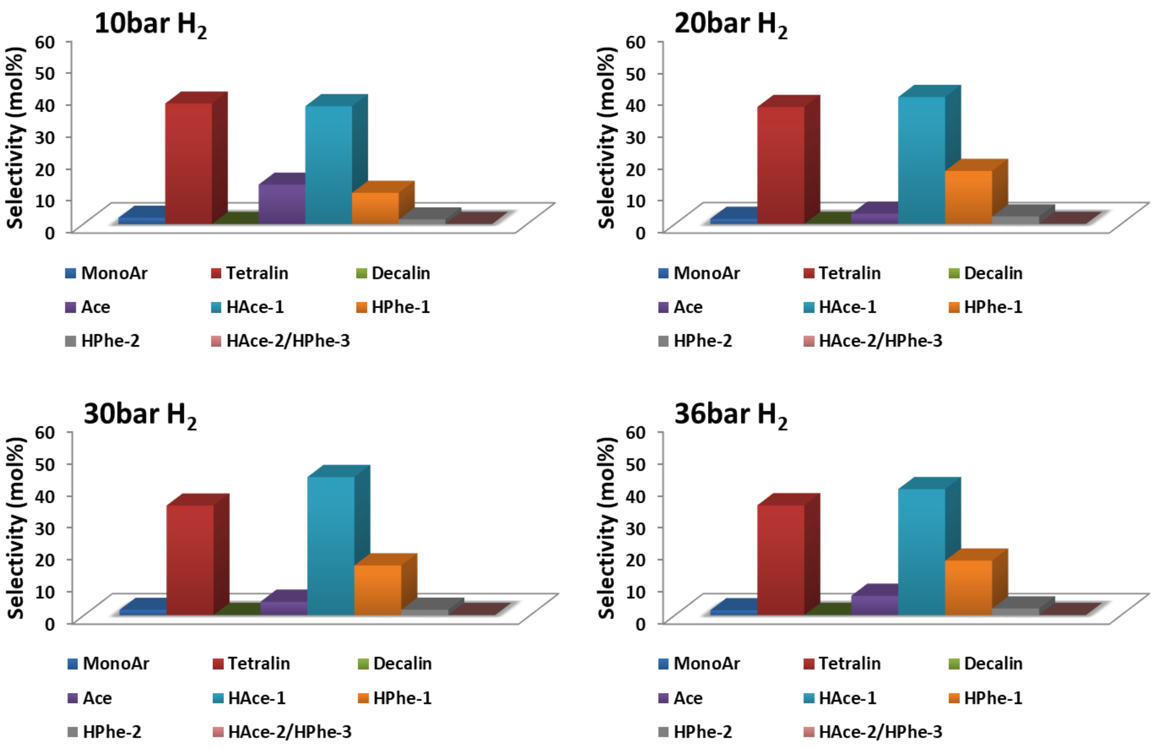

Figure S11. Selectivity to the different groups of products (compared at ≈53% conversion) for 1.3wt%Pd/TiO_2 Nano catalyst by using different H_2 pressures. <u>Reaction conditions</u>: 0.5 g of tars-type compounds, 4 g of n-hexadecane, 0.2 g of catalyst, at 275 °C during 7 h.

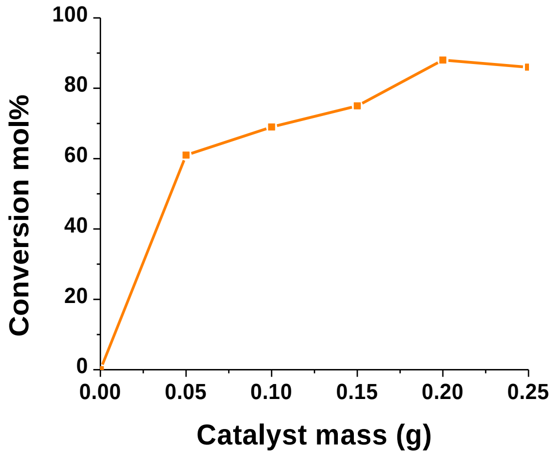

Figure 12. Catalyst loading optimization in tars mild hydrotreatment over 1.3wt%Pd/TiO_2 Nano. Reaction conditions: 0.5 g of tars-type compounds, 4 g of n-hexadecane, at 275 °C and 30 bar of H_2 during 7 h.

Reusability tests

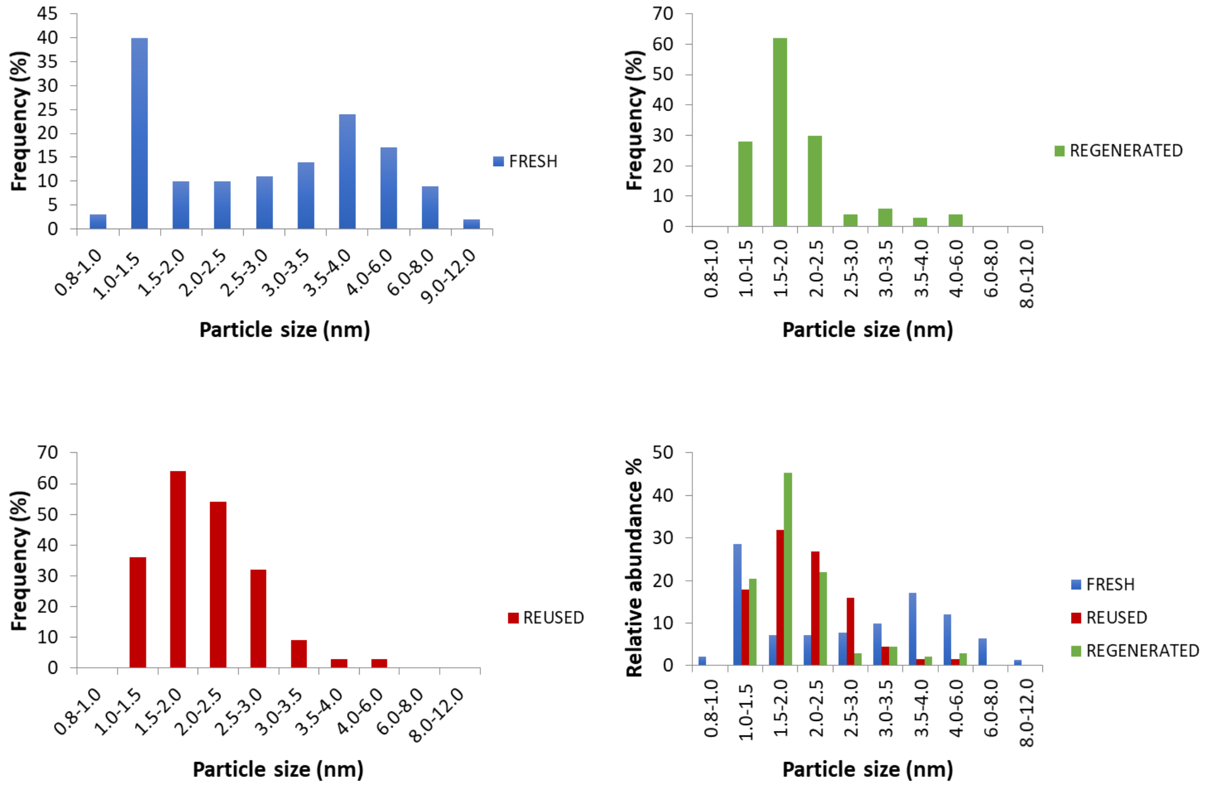

Figure S13. Pd particle size distribution of fresh, reused and regenerated samples of Pd/TiO_2 Nano catalyst.

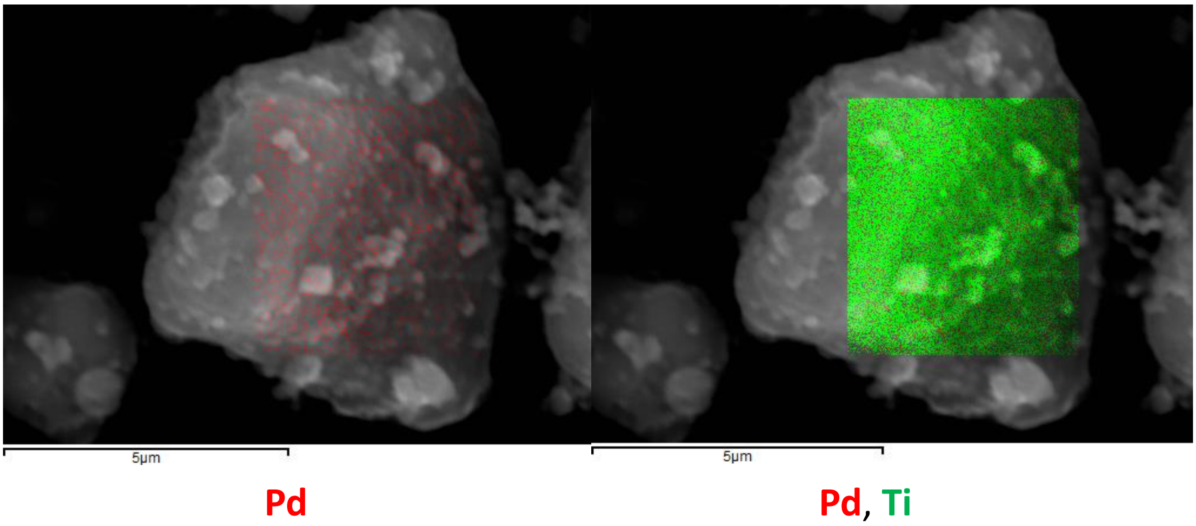

Figure S14. SEM-EDX for the fresh sample of Pd/TiO_2 Nano catalyst.

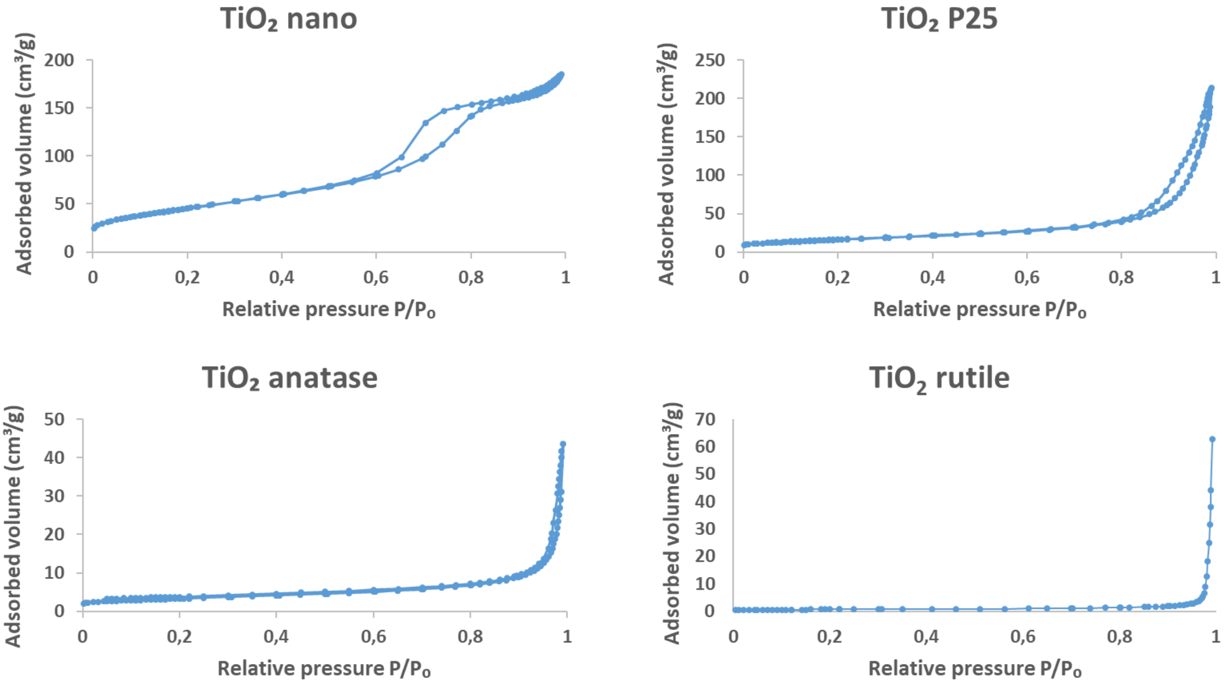

Figure S15. Nitrogen adsorption-desorption isotherms for different TiO_2 materials.

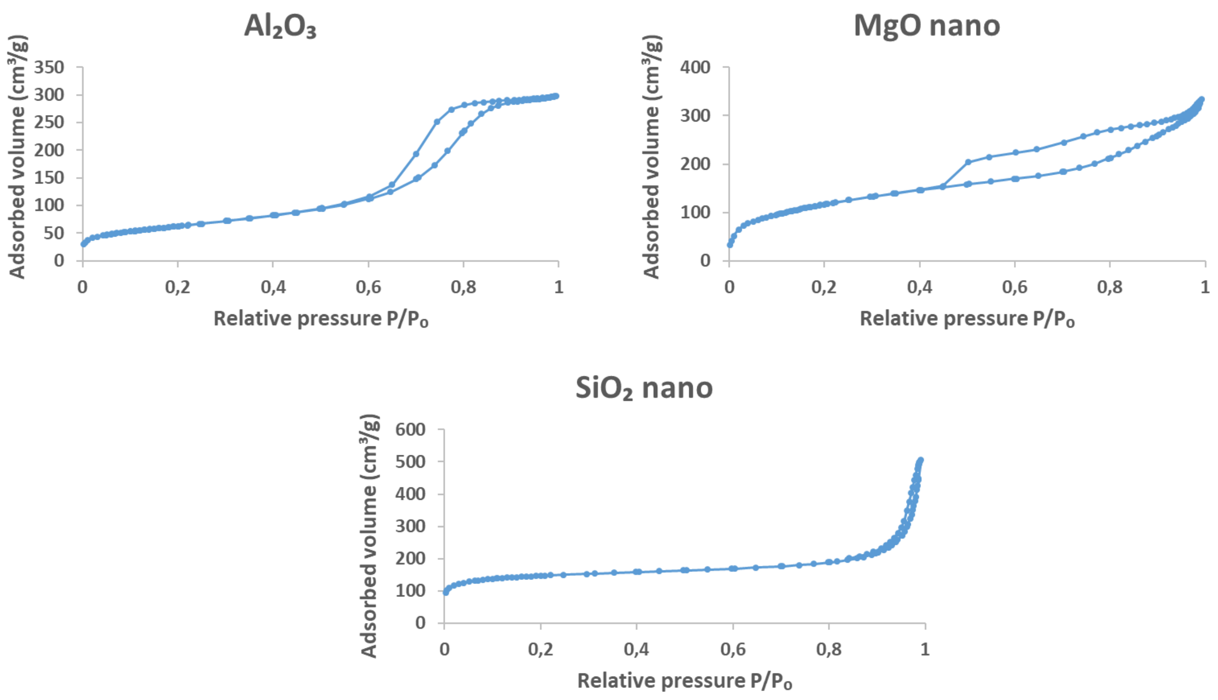

Figure S16. Nitrogen adsorption-desorption isotherms for different metal oxides materials.
